# Which Diet-Related Behaviors in Childhood Influence a Healthier Dietary Pattern? From the Ewha Birth and Growth Cohort

**DOI:** 10.3390/nu9010004

**Published:** 2016-12-23

**Authors:** Hye Ah Lee, Hyo Jeong Hwang, Se Young Oh, Eun Ae Park, Su Jin Cho, Hae Soon Kim, Hyesook Park

**Affiliations:** 1 Department of Preventive Medicine, School of Medicine, Ewha Womans University, Seoul 07985, Korea; 2 Biomaterials Research Institute, Sahmyook University, Seoul 01795, Korea; fullmoon0118@naver.com; 3 Department of Food & Nutrition, Research Center for Human Ecology, College of Human Ecology, Kyung Hee University, Seoul 02447, Korea; seyoung@khu.ac.kr; 4 Department of Pediatrics, School of Medicine, Ewha Womans University, Seoul 07985, Korea; pea8639@ewha.ac.kr (E.A.P.); sujin-cho@ewha.ac.kr (S.J.C.); hyesk@ewha.ac.kr (H.S.K.)

**Keywords:** children, dietary pattern, diet-related behavior, longitudinal study

## Abstract

This study was performed to examine how childhood dietary patterns change over the short term and which changes in diet-related behaviors influence later changes in individual dietary patterns. Using food frequency questionnaire data obtained from children at 7 and 9 years of age from the Ewha Birth and Growth Cohort, we examined dietary patterns by principal component analysis. We calculated the individual changes in dietary pattern scores. Changes in dietary habits such as eating a variety of food over two years were defined as “increased”, “stable”, or “decreased”. The dietary patterns, termed “healthy intake”, “animal food intake”, and “snack intake”, were similar at 7 and 9 years of age. These patterns explained 32.3% and 39.1% of total variation at the ages of 7 and 9 years, respectively. The tracking coefficient of snack intake had the highest coefficient (γ = 0.53) and that of animal food intake had the lowest (γ = 0.21). Intra-individual stability in dietary habits ranged from 0.23 to 0.47, based on the sex-adjusted weighted kappa values. Of the various behavioral factors, eating breakfast every day was most common in the “stable” group (83.1%), whereas consuming milk or dairy products every day was the least common (49.0%). Moreover, changes in behavior that improved the consumption of milk or dairy products or encouraged the consumption of vegetables with every meal had favorable effects on changes in healthy dietary pattern scores over two years. However, those with worsened habits, such as less food variety and more than two portions of fried or stir-fried food every week, had unfavorable effects on changes in healthy dietary pattern scores. Our results suggest that diet-related behaviors can change, even over a short period, and these changes can affect changes in dietary pattern.

## 1. Introduction

To improve diet, understanding how dietary patterns develop is important in epidemiological studies related to chronic diseases and public health planning [1]. The critical period for the development of certain dietary patterns, during which time the development should be tracked, remains a major issue in nutritional epidemiology. Several studies have suggested that dietary patterns are determined in childhood [2,3,4]. One large prospective cohort study, the Avon Longitudinal Study of Pregnancy and Childhood (ALSPAC), indicated that the dietary pattern at 7 years old was a determinant of later dietary patterns based on the results of tracking coefficients from diverse statistical approaches [4]. However, previous studies focusing on the stability of dietary patterns yielded mixed results with only moderate [5,6] or slight tracking [1,7].

With regard to the critical period, children learn what, when, and how to eat through direct experience observing others [8]. Thus, it is important to identify critical intervention factors to suggest appropriate strategies for improving dietary behaviors. However, the effectiveness of interventions to modify dietary behaviors remains unclear [9,10]. One recent observational study from the NEXT Generation Health Study among American teens indicated within-individual correlations of 41%–51% in food group intake and meal practices over four years. It was also reported that time-varied frequencies of intake of fruit/vegetables or snacks were associated with time-varied meal practices, such as the frequency of fast food intake [6]. However, this study focused on intakes of specific food groups or eating behaviors.

Childhood dietary patterns could be reflected in underlying food preferences, diet-related behaviors, as well as environmental factors, such as household income and parental education level [8]. Studying dietary patterns is a reasonable approach because the net effect of a single food or nutrient cannot be separated from the total. Several methodologies have been introduced to explore dietary patterns [11,12]. Of these, principal component analysis (PCA) is a multidimensional reduction analysis method to examine the correlations of food intakes, and is commonly used in nutritional epidemiology [13]. Several studies using PCA reported several dietary patterns in children and adolescents as “healthy”, “traditional”, “Western”, and “junk or processed food intakes”, among others [5,14,15]. However, Hu suggested that much more research is necessary in diverse populations due to sociocultural differences [12]. In addition, many previous studies did not take into consideration changes in dietary pattern or related behaviors. A better understanding of changes in diet-related behaviors and dietary patterns may provide an opportunity to explore appropriate intervention strategies.

Using data from a Korean cohort study, we evaluated how childhood dietary patterns change in the short term, and which changes in diet-related behavior influence later changes in individual dietary patterns.

## 2. Methods

### 2.1. Study Subjects

This study was part of an ongoing Ewha Birth and Growth Cohort study by the Ewha Woman’s University Mokdong Hospital, Seoul, Korea. It was established to longitudinally evaluate the growth and health of children, and it commenced in the early life of the subjects. Briefly, mothers (*n* = 940) were enrolled in the study between 2001 and 2006 during prenatal care visits when they were 24–28 weeks pregnant, and a follow-up was done with their children 3, 5, and 7 years later. About 30% of all possible subjects agreed to participate in the study [16]. A detailed description of the cohort composition, including methodology, has been published elsewhere [17]. Through follow-up at 7 or 9 years of age, a diet-related questionnaire survey was performed using a food frequency questionnaire (FFQ) and questions related to dietary habits. Follow-up at 7 years of age began in 2009, but data were collected using FFQs from 2010. A total of 364 and 380 children participated in follow-up at 7 years (follow-up years from 2009 to 2014) and 9 years of age (follow-up years from 2011 to 2015), respectively. Of these, completed FFQs were obtained for 279 and 360 children, respectively. FFQ data for both follow-up times were obtained for 154 children. Approximately 41.9% of cohort subjects were lost to follow-up (they changed their telephone numbers or withdrew) at the time of the 7-year follow-up, and an additional 3.4% were lost to follow-up at 9 years. Written informed consent for participation in the study was obtained from the parents or guardians of all study participants at the time of follow-up. The study protocol was approved by the Institutional Review Board of the Ewha Womans University Hospital.

### 2.2. Dietary Data and Dietary Pattern Analysis

Individual dietary data for the past year were collected by the parents or guardians and validated by trained interviewers using the FFQ (90 food items). Both the reproducibility (*r* value = 0.5–0.8) and validity (*r* value = 0.3–0.6) of the instrument were acceptable, as reported elsewhere [18,19]. We used the same questionnaire at both follow-ups. These food items were placed into nine non-overlapping categories according to the frequency of consumption, ranging from “rarely eaten” to “more than three times per day” during the preceding year, and portion size, namely, small, average, or large. In this study, we used food intake frequencies to construct dietary patterns [20]. Weekly intake from the FFQ was calculated by multiplying the consumption frequency of each food by the following values for each frequency option: never = 0; once a month = 0.23; two-to-three times a month = 0.58; one-to-two times a week = 1.5; three-to-four times a week = 3.5; five-to-six times a week = 5.5; once a day = 7; twice a day = 14; and three times a day = 21. Of the 90 food items, similar items were grouped to form 22 food groups (Table S1). Prior to PCA, data were standardized using means and standard deviations, and the dietary patterns at each time point were analyzed via PCA with varimax rotation. The first three components were appropriate, based on the screen plots and eigenvalues ≥1. The factor-loading values by dietary pattern are shown in Table 1. Factors with loading >0.3 were considered the principal contributors to a dietary pattern [5]. Factor scores were used as outcomes, which were defined as dietary pattern scores. To assess changes in the same dietary patterns over time, we calculated the z scores of food group intake from children aged 9 years using the means and standard deviations obtained when they were 7 years of age. These were multiplied by factor-loading values for each dietary pattern (it was obtained using the data for 7 years old), and then summed. This approach has been used in previous studies [5,21].

### 2.3. Dietary Habits

We collected data regarding dietary habits using the following questions:

DH1. Do you eat more than two servings of milk or dairy products every day?
DH2. Do you eat meat, fish, egg, beans, or tofu with every meal?
DH3. Do you eat vegetables other than kimchi with every meal?
DH4. Do you eat one serving size of fruit or drink one portion of fruit juice every day?
DH5. Do you eat more than two servings of fried or stir-fried food every week?
DH6. Do you eat more than two servings of fatty meat (e.g., bacon, ribs, eel) every week?
DH7. Do you generally add table salt or soy sauce to food?
DH8. Do you eat three regular meals per day?
DH9. Do you eat ice cream, cake, snacks, and soda (e.g., cola, cider) as snacks more than twice a week?
DH10. Do you eat a variety of food every day?

The possible responses were “always”, “generally”, and “seldom”. Questions DH1–4, DH8, and DH10 evaluated healthy dietary habits, and questions DH5–7 and DH9 evaluated unhealthy dietary habits. This mini-dietary assessment tool has been validated in previous studies [22,23]. In addition, the subjects were also asked “Do you eating breakfast every day?” to which they responded either “yes” or “no”. Changes in individual behaviors were classified as “increased”, “stable”, or “decreased”. If one subject at 7 years old replied “seldom” to the question “Do you eat over two servings of milk or dairy products every day?” and answered “always” or “generally” to the same question at 9 years old, the behavior was defined as “increased”, while the opposite was classified as “decreased”. Finally, those who gave the same answer at both follow-ups were defined as “stable”.

### 2.4. Other Variables

We also evaluated data on household income, parental education, parental obesity, time spent watching television (TV), and child body mass index (BMI); previous studies have shown that these were potentially important factors [2,6,15,21]. Monthly household income was grouped as “low” (<3 million South Korean Won (KRW)), “middle” (3.0–4.9 million KRW), or “high” (>5 million KRW). Parental education level was classified into two levels (graduated from high school; some college or higher). Parental obesity was defined as BMI ≥ 25 kg/m^2^, calculated by dividing weight by height squared. These data were collected by a self-reported questionnaire at follow-up. The daily amount of time spent watching TV was categorized as <1 h, 1–2 h, and >2 h. Child BMI was calculated by measuring height and weight at both follow-ups.

### 2.5. Statistical Analysis

The associations between dietary pattern scores and socioeconomic factors, parental factors, and dietary habits were analyzed using the *t* test or analysis of variance (ANOVA). Based on the findings from the univariate analyses, we selected potentially significant factors (*p* < 0.2) for inclusion in the multiple regression analyses. A factor was considered relevant if it was potentially related to any dietary pattern. However, paternal education was not considered, being strongly associated with household income (an indicator of socioeconomic status). In multiple regression analysis, responses to dietary habits were treated as continuous variables (e.g., “always” = 2, “generally” = 1 and “seldom” = 0 for questions related to healthy dietary habits and applied in reverse for questions about unhealthy dietary habits) by considering multicollinearity. Multicollinearity in multiple regression was assessed based on variance inflation factors and it had a value <2 across our results. Correlations between dietary pattern scores at the two time points were estimated using Spearman’s correlation, and the changes in dietary pattern scores within an individual were assessed using the paired *t* test. To determine the changes in dietary habits, we used weighted kappa and proportion of dietary habit changes stratified according to sex. The independent effects of changes in individual behaviors were expressed as “increased”, “stable”, or “decreased” over time in terms of changes in dietary patterns after taking sex, household income, and other parameters, into consideration. The change in watching TV was excluded due to data on this variable being missing for a large proportion of the subjects (13.6%). In all analyses, *p* < 0.05 (two-tailed test) was taken to indicate statistical significance. All statistical analyses were conducted using SAS 9.3 (SAS Institute, Cary, NC, USA).

## 3. Results

With regard to the characteristics of the study subjects, about half were boys (49.46%) with an average BMI of 15.95 kg/m^2^ (95% confidence interval: 15.71–16.20 kg/m^2^). Most of the children ate breakfast daily (84.84%). Of all of the children, 41.73% watched television for more than 2 h per day. In terms of household income (an indicator of socioeconomic status), 20.59%, 41.54%, and 37.87% of children were in the low, middle, and high groups, respectively. Table 1 shows dietary patterns derived from PCA at each time point. The first three components accounted for 32.27% (PC1: 13.83%, PC2: 10.23%, and PC3: 8.21% at 7 years old) and 39.10% (PC1: 15.12%, PC2: 16.20%, and PC3: 7.77% at 9 years old) of total variation. The three components were referred to as “healthy intake”, “animal food intake”, and “snack intake”. Healthy intake was positively associated with vegetable and bean items. Animal food intake showed weighted loading factors in meat and fish items. Finally, snack intake showed positive loading factors in candy, soda, and bread items. The patterns were similar at the older age, but some food types had more weighted loading factors. Healthy intake at 9 years of age showed more weighted loading factors in fruit, milk, nut, and seaweed food groups than at the younger age.

The results of univariate association are presented in Table S2. Higher household income status tended to show higher mean health intake pattern scores at 7 years of age. In addition, healthy intake was significantly associated with eating breakfast every day and all of the related healthy dietary habits. Animal food intake was associated with sex, eating fatty meat, and generally adding table salt or soy sauce to food. Subjects that spent a longer time watching TV had higher mean snack pattern scores. Snack intake also showed a significant association with eating milk or dairy products; eating fruit or drinking fruit juice every day; eating fried or stir-fried food; generally adding table salt or soy sauce to food; and eating ice cream, cake, snacks, and soda (e.g., cola, cider) as snacks.

In multiple regression analysis, eating breakfast every day and eating a variety of food every day showed independent effects on the healthy pattern with positive coefficients (*β* = 0.24, *β* = 0.19, respectively). With regard to animal food intake, female gender showed higher pattern scores, while unusual behaviors with regard to fatty meat and generally adding table salt or soy sauce to food showed lower pattern scores. Pattern scores in snack intake were also positively associated with watching TV (*β* = 0.15) and negatively associated with eating vegetables other than kimchi (*β* = −0.23). Moreover, several factors showed independent effects at both follow-up times. Eating a variety of food was consistently associated with healthy intake at both follow-up times. Eating vegetables other than kimchi with every meal was also negatively associated with snack intake. Otherwise, there were no significant associations with animal food intake (Table 2).

Table 3 shows the results regarding changes in dietary pattern scores and tracking coefficients of dietary patterns. The tracking coefficient of snack intake showed the highest coefficient (0.53, *p* < 0.0001) and animal food intake showed the lowest coefficient (0.21, *p* < 0.01). The mean dietary pattern scores from the earlier time point showed increasing tendencies across dietary patterns, and this score was highest for animal food intake (Δ = 0.20, *p* < 0.001).

**Figure 1:**
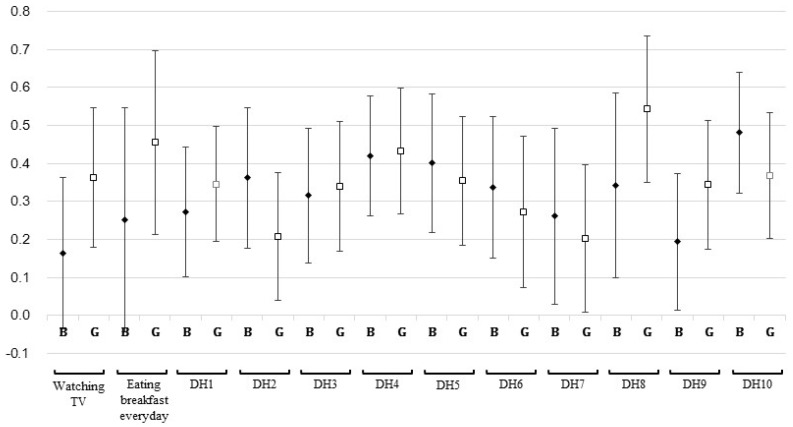
Weighted *κ* of two repeated measures for behaviors by sex. B: boys (black diamonds), G: girls (white squares), line indicates 95% confidence interval. DH1: Eating more than two portions of milk or dairy products every day. DH2: Eating meat, fish, egg, beans, or tofu with every meal. DH3: Eating vegetables other than kimchi with every meal. DH4: Eating one portion of fruit or drinking one portion of fruit juice every day. DH5: Eating more than two portions of fried or stir-fried food every week. DH6: Eating more than two portions of fatty meat (e.g., bacon, ribs, eel) every week. DH7: Generally adding table salt or soy sauce to food. DH8: Eating three regular meals per day. DH9: Eating ice cream, cake, snacks, and soda (e.g., cola, cider) as snacks more than twice a week. DH10: Eating a variety of food every day. The possible responses to dietary habits (DHs) were “always”, “generally”, or “seldom”. The daily TV-watching time was categorized as <1 h, 1–2 h and >2 h. Eating breakfast everyday was grouped as yes or no.

Figure 1 shows the intra-individual stability of dietary habits over two years by sex. The weighted kappa values of eating breakfast every day, watching TV, eating three regular meals a day, and eating ice cream, cake, snacks, and soda were markedly higher in girls than in boys, while those of eating a variety of food every day and eating meat, fish, egg, beans, or tofu with every meal were higher in boys than in girls. Sex-adjusted weighted kappa values ranged from 0.23 to 0.47. Of the behavior factors, eating breakfast every day showed the highest proportion for “stable” (83.1%), while eating milk or dairy products every day showed the lowest proportion (49.0%) (Table 4).

Table 5 shows the effects of behavioral changes on changes in dietary patterns. Those with improved dietary habits (who ate vegetables other than kimchi with every meal and consumed more than two portions of milk or dairy products every day) exhibited improved healthy intake pattern scores over two years, whereas those with worsening habits (less food variety and more than two portions of fried or stir-fried food every week) exhibited decreased scores. In addition, worsening with regard to eating ice cream, cake, snacks, and soda as snacks increased in the animal food intake patterns. However, other dietary habit changes were not significantly related to dietary pattern changes.

## 4. Discussion

We explored the childhood dietary patterns at 7 and 9 years of age and assessed the changes in individual dietary patterns. There were three dietary patterns, namely, “healthy intake”, “animal food intake”, and “snack intake”, the contributions of which differed at each time point. The tracking coefficients ranged from 0.21 for animal food intake to 0.53 for snack intake. Overall, the mean dietary pattern scores tended to increase over time. Moreover, changes in behaviors that improved the consumption of milk and dairy products or vegetables with every meal exhibited improved healthy intake pattern scores over two years, whereas those with worsened habits, such as less food variety and more than two portions of fried or stir-fried food every week, exhibited decreased scores.

Individual dietary patterns change over time, even in childhood [5,21]. As individual diet-related behaviors can also change, the above findings appear reasonable. Supporting evidence from intervention studies is required to improve the dietary habits of children. Repeated measures in cohort design could also be used to assess the effects of natural changes in dietary behaviors. In this study, we examined the effects of changes in diet-related behaviors on changes in pattern scores. The results indicated that improved individual dietary behaviors related to eating vegetables with every meal independently attributed to increased healthier dietary patterns over time. The advantages of eating vegetables have been demonstrated by several systematic review studies with regard to diverse health effects, as well, they reflect the nutritional quality of meals [24,25,26]. However, interventions involving increasing the vegetable intake among children were unsuccessful [10]. One large study conducted in American teens estimated that within-person correlations for eating behaviors were >0.41 for the intake frequencies of fruit and vegetables, whole grains, soda, and snacks using four sets of repeated-measure data, and time-varying intake frequency of fruit and vegetables was positively associated with time-varying breakfast and family meals and negatively associated with fast food intake using a generalized estimating equations model. However, this previous study did not discuss the intake of various food [6]. In addition, eating a variety of food reflects adequate intake of essential nutrients, and is recommended in most dietary guidelines, including those in South Korea [27]. In addition, milk and dairy products are major sources of calcium for growing children. A national study found that more than half of all Korean children have inadequate calcium intake [28]. Although it is difficult to compare previous findings with ours because different assessment methods were used, we also found that it was beneficial to eat a variety of foods including milk and dairy products. In contrast, fried or stir-fried food was associated with high fat intake. We found that increased consumption of fried or stir-fried food unfavorably influenced a healthy dietary pattern. Thus, our results are meaningful in terms of epidemiological approaches. The unexpected results regarding the association between changes in eating ice cream, cake, snacks, and soda (e.g., cola, cider) as snacks seemed to be influenced by increased food frequency. Unlike other dietary habits, overall intake frequencies were higher for subjects with worsening behaviors regarding eating ice cream, cake, snacks, and soda as snacks compared with those with stable and increased behaviors. Indeed, all dietary pattern scores increased with a decrease of that behavior, as shown in Table 5.

As presented in Table 1, there were some changes in the composition of the three types of dietary patterns over two years. This was not surprising based on the results of previous studies [5,7]. Generally, the traditional diet in South Korea includes high levels of various vegetables and is low in fat [29,30]. These features were similar in our study. A previous study among South Korean adults also showed similar patterns to those in the present study [30], but little evidence was available regarding dietary patterns in South Korean children. For variation during two years, the intakes of most food items increased at 9 years old compared to those at 7 years old, with the exceptions of yellow vegetables, mushrooms, fruit, milk, and seaweed laver. Soda, potatoes, bread, and pizza showed relatively large increases (data not shown). Increased soda intake with increasing age was also observed in a previous growth and health study from the National Heart, Lung, and Blood Institute [31] and the Bogalusa Heart Study [32]. The environmental influences on food choice, preference, and accessibility vary over the lifespan. The opportunity to choose food oneself would increase with increasing age. Consistent with our study, the results regarding dietary pattern derived from the ALSPAC study showed that notable loading factors differed among food groups at 9 years old compared with those at 7 years old [4]. Another two studies using reduced rank regression and cluster analysis of the same cohort data indicated that dietary patterns at 7 years of age was a strong determinant of later dietary patterns [4,21]. Children starting school and participating in various activities are placed in a new environment, which may influence food choice. Therefore, further studies on changes in dietary habits and patterns over longer periods are required.

Several factors potentially related to dietary patterns have been reported, including low maternal education level [14,15,21]; socioeconomic status [15]; passive smoking and watching TV [14]; childhood obesity or maternal obesity [2]; diet-related factors, such as being vegetarian [33]; and TV meals, family meals, and breakfast [6]. However, most of these studies reported effects at a critical time on dietary patterns rather than changes in potential factors as mentioned above. Parental education level and birth- or infancy-related features were considered as time-independent factors. In this study, associations between household income and dietary patterns were more notable than parental education level. These observations may be explained by more highly educated parents having a better understanding of nutritional information and being more likely to restrict their children’s intake of unhealthy food [34]. These observations may also be explained by the dependence of the ability to pay for food or groceries on household income, because a nutrient-dense diet is more expensive than an energy-dense diet [35]. However, any independent effects of household income were not significant as determined by multiple regression analyses. Moreover, we found no association with maternal obesity and child BMI.

Several points must be taken into consideration when interpreting the results of our study. Our results were derived from a smaller sample than previous studies. Bias due to follow-up loss would probably have an impact on the results. However, there were no differences in the distribution of demographic factors or dietary habits between subjects who successfully followed up or were lost to follow-up, except in eating a variety of food (*p*_chi_ = 0.02). Therefore, this did not seem to affect our results. To allow comparison, loading factors were applied to the calculated dietary pattern scores at the older age. This approach also has limitations in that it did not reflect the changes in characteristics of dietary patterns. Healthy intake pattern showed a positive loading factor for eating vegetables at the two time points, but eating fruit showed a higher weighting for a healthy diet at 9 years old. Thus, scores of a change within dietary patterns do not reflect the above-mentioned change. We used the same validated questionnaires at both follow-ups, and all of the data were collected by trained dieticians. Thus, any bias imparted by this procedure would be small. Moreover, there could be residual confounding effects of several factors that were not considered in this study.

This cohort study had several strengths. In this cohort study, we observed behavioral changes within individuals and were able to assess the associated effects, although the observational period was short. This work is the first step towards observationally determining whether behavioral changes in early life can modify dietary patterns, thereby improving health later in life. In addition, our results yield important data from a non-Western country. Future studies are needed to determine if the effects that we observed will persist in the long term.

## 5. Conclusions

Our results suggest that single measurements of food frequency intake and dietary habits during childhood may be insufficient to determine individual dietary patterns. In addition, diet-related behaviors can change, even in a short period, and such changes can affect dietary patterns.

## Figures and Tables

**Table 1:** Factor loading scores for the first three components derived from principal component analysis.

	Healthy Intake		Animal Food Intake		Snack Intake	
	7 Years	9 Years	7 Years	9 Years	7 Years	9 Years
Variance	13.83%	15.12%	10.23%	16.20%	8.21%	7.77%
Yellow vegetables	0.840	0.820	0.070	0.108	−0.020	0.050
Green vegetables	0.800	0.795	0.149	0.105	0.045	0.074
White vegetables	0.475	0.417	0.268	0.014	−0.040	0.057
Mushrooms	0.802	0.677	−0.066	0.081	−0.037	0.126
Beans	0.476	0.522	0.181	0.089	0.198	0.091
Potatoes	0.280	0.468	0.071	0.056	0.227	0.277
Fruit	0.271	0.341	0.160	0.038	0.151	0.148
Nuts	0.222	0.418	0.200	0.090	0.044	0.150
Shellfish	0.106	0.019	0.798	0.948	0.073	0.047
White fish	0.092	0.009	0.714	0.938	0.152	0.022
Blue fish	0.144	0.200	0.598	0.909	0.257	0.063
Meat	0.444	0.081	0.587	0.853	0.125	0.239
Eggs	0.276	0.136	0.120	0.400	0.268	−0.007
Rice	0.066	0.089	0.224	−0.037	0.063	0.072
Bread	0.108	0.078	−0.023	0.033	0.712	0.399
Jam	−0.006	0.192	0.023	0.005	0.617	0.396
Soda	0.047	0.226	0.094	0.024	0.394	0.559
Milk	0.235	0.388	0.028	0.065	0.346	0.459
Candy	−0.047	0.048	0.109	0.011	0.339	0.509
Pizza	−0.030	0.042	0.240	0.059	0.322	0.424
Noodles	0.054	0.070	0.175	0.075	0.251	0.433
Seaweed	0.089	0.529	0.138	0.083	0.234	0.127

**Table 2:** Multiple regression analysis of the effects of potential factors on dietary pattern at two observation times.

Potential Factor at 7 Years	Dietary Pattern Scores at 7 Years Old						Dietary Pattern Scores at 9 Years Old					
	Healthy Intake		Animal Food Intake		Snack Intake		Healthy Intake		Animal Food Intake		Snack Intake	
	*β*	S.E.	*β*	S.E.	*β*	S.E.	*β*	S.E.	*β*	S.E.	*β*	S.E.
Sex	−0.010	0.06	0.183 ^a^	0.06	−0.078	0.10	−0.027	0.10	0.056	0.13	0.127	0.18
Monthly household income	0.023	0.04	0.003	0.04	0.014	0.07	0.037	0.07	0.068	0.09	0.186	0.13
Body mass index (BMI)	−0.007	0.02	−0.0002	0.02	−0.016	0.03	−0.011	0.03	−0.021	0.04	0.005	0.05
Maternal obesity	0.007	0.08	−0.002	0.08	−0.222	0.14	−0.051	0.14	0.022	0.18	0.007	0.24
Watching television (TV)	−0.043	0.04	0.057	0.04	0.152 ^a^	0.07	−0.004	0.07	−0.052	0.09	0.015	0.13
Eating breakfast every day	0.242 ^a^	0.10	−0.071	0.10	−0.071	0.17	0.091	0.16	0.206	0.20	−0.296	0.28
Healthy dietary habits												
DH1	0.071	0.05	0.036	0.05	0.129	0.08	0.026	0.07	−0.0004	0.09	0.152	0.13
DH2	−0.002	0.05	0.071	0.05	0.016	0.09	−0.048	0.09	0.007	0.11	−0.114	0.16
DH3	0.068	0.05	0.026	0.05	−0.226 ^a^	0.08	−0.153	0.08	−0.067	0.10	−0.435 ^a^	0.14
DH4	0.048	0.05	−0.043	0.05	0.117	0.09	0.154	0.08	−0.120	0.10	0.028	0.14
DH8	0.075	0.06	0.069	0.06	−0.069	0.11	−0.083	0.10	0.069	0.13	0.063	0.18
DH10	0.190 ^a^	0.05	0.047	0.05	0.114	0.08	0.166 ^a^	0.07	−0.021	0.09	0.123	0.13
Unhealthy dietary habits												
DH5	0.064	0.05	0.021	0.05	−0.047	0.08	−0.129	0.07	−0.035	0.09	−0.245	0.13
DH6	−0.038	0.05	−0.154 ^a^	0.05	−0.105	0.08	−0.047	0.08	−0.048	0.11	0.250	0.15
DH7	−0.005	0.06	−0.127 ^a^	0.06	−0.152	0.10	−0.027	0.10	−0.052	0.12	−0.358 ^a^	0.17
DH9	0.010	0.04	−0.007	0.04	−0.113	0.07	0.006	0.07	0.024	0.08	−0.160	0.12

^a^ *p* < 0.05. S.E. = standard error. DH1: Eating more than two portions of milk or dairy products every day. DH2: Eating meat, fish, eggs, beans, or tofu with every meal. DH3: Eating vegetables other than kimchi with every meal. DH4: Eating one portion of fruit or drinking one portion of fruit juice every day. DH5: Eating more than two portions of fried or stir-fried food every week. DH6: Eating more than two portions of fatty meat (e.g., bacon, ribs, eel) every week. DH7: Generally adding table salt or soy sauce to food. DH8: Eating three regular meals a day. DH9: Eating ice cream, cake, snacks, and soda (e.g., cola, cider) as snacks more than twice a week. DH10: Eating a variety of food every day. The possible responses to dietary habits (DHs) were “always”, “generally”, or “seldom”. The daily TV-watching time was categorized as <1 h, 1–2 h, and >2 h. Eating breakfast everyday was grouped as yes or no.

**Table 3:** Changes in dietary pattern scores between the two observational times.

Dietary Pattern Scores	Tracking Coefficient ^†^	At 7 Years		At 9 Years ^‡^		Differences of Dietary Pattern Scores ^‡^		Paired *t* Test *p*
		Mean	S.D.	Mean	S.D.	Mean	S.D.	
Healthy intake	0.369 ^a^	−0.106	0.498	0.062	0.613	0.176	0.624	<0.001
Animal food intake	0.215 ^b^	−0.091	0.479	0.123	0.691	0.204	0.717	<0.001
Snack intake	0.526 ^a^	−0.003	0.861	0.213	0.969	0.161	0.864	0.02

^†^ The tracking coefficients between the dietary pattern scores at the two time points were estimated by deriving Spearman’s correlations. ^‡^ Results are presented for those who participated in both follow-ups (*n* = 154). ^a^ *p* < 0.0001, ^b^ *p* < 0.01. S.D. = standard deviation.

**Table 4:** Changes in individual’s behaviors over two years.

	Sex-Adjusted Weighted Kappa	Stable		Increased		Decreased	
		*n*	%	*n*	%	*n*	%
Watching TV	0.271	75	56.39	27	20.30	31	23.31
Eating breakfast	0.373	128	83.12	9	5.84	17	11.04
Healthy dietary habits							
DH1	0.314	75	49.02	30	19.61	48	31.37
DH2	0.277	82	53.95	34	22.37	36	23.68
DH3	0.328	83	54.25	39	25.49	31	20.26
DH4	0.427	90	59.21	25	16.45	37	24.34
DH8	0.466	115	75.66	18	11.84	19	12.5
DH10	0.427	88	57.52	42	27.45	23	15.03
Unhealthy dietary habits							
DH5	0.376	90	59.21	27	17.76	35	23.03
DH6	0.307	97	63.4	27	17.65	29	18.95
DH7	0.227	101	66.01	25	16.34	27	17.65
DH9	0.273	74	48.68	41	26.97	37	24.34

DH1: Eating more than two portions of milk or dairy products every day. DH2: Eating meat, fish, egg, beans, or tofu with every meal. DH3: Eating vegetables other than kimchi with every meal. DH4: Eating one portion of fruit or drinking one portion of fruit juice every day. DH5: Eating more than two portions of fried or stir-fried food every week. DH6: Eating more than two portions of fatty meat (e.g., bacon, ribs, eel) every week. DH7: Generally adding table salt or soy sauce to food. DH8: Eating three regular meals per day. DH9: Eating ice cream, cake, snacks, and soda (e.g., cola, cider) as snacks more than twice a week. DH10: Eating a variety of food every day. The possible responses to dietary habits (DHs) were “always”, “generally”, or “seldom”. The daily TV-watching time was categorized as <1 h, 1–2 h, and >2 h. Eating breakfast everyday was grouped as yes or no.

**Table 5:** Effects of behavioral changes over two years within dietary patterns.

		Difference in Dietary Pattern 1 Score		Difference in Dietary Pattern 2 Score		Difference in Dietary Pattern 3 Score	
		*β*	S.E.	*β*	S.E.	*β*	S.E.
Eating breakfast	increased	−0.057	0.23	−0.483	0.29	0.251	0.35
	decreased	−0.175	0.17	−0.375	0.23	−0.013	0.27
Healthy dietary habits							
DH1	increased	0.374 ^a^	0.15	0.122	0.20	0.240	0.23
	decreased	0.143	0.13	0.255	0.17	0.130	0.20
DH2	increased	−0.089	0.14	0.152	0.18	0.060	0.21
	decreased	−0.215	0.14	−0.233	0.17	−0.134	0.21
DH3	increased	0.272 ^a^	0.13	−0.136	0.17	0.278	0.20
	decreased	0.167	0.15	0.078	0.19	0.263	0.23
DH4	increased	−0.073	0.15	0.128	0.20	0.021	0.24
	decreased	−0.196	0.13	−0.042	0.17	−0.174	0.20
DH8	increased	−0.108	0.17	0.141	0.23	−0.140	0.26
	decreased	0.037	0.17	0.235	0.22	−0.073	0.25
DH10	increased	0.117	0.13	0.099	0.17	0.090	0.20
	decreased	−0.301 ^a^	0.15	0.021	0.20	−0.285	0.23
Unhealthy dietary habits							
DH5	increased	0.153	0.14	0.046	0.19	0.219	0.22
	decreased	−0.310 ^a^	0.14	−0.199	0.18	−0.261	0.22
DH6	increased	0.095	0.15	−0.090	0.19	−0.176	0.22
	decreased	−0.070	0.14	0.166	0.19	−0.033	0.22
DH7	increased	−0.137	0.16	−0.287	0.20	−0.081	0.23
	decreased	−0.051	0.14	0.009	0.18	0.047	0.22
DH9	increased	−0.010	0.13	−0.227	0.17	0.019	0.20
	decreased	0.239	0.14	0.390 ^a^	0.18	0.348	0.21

^a^ *p* < 0.05. DH1: Eating more than two portions of milk or dairy products every day. DH2: Eating meat, fish, egg, beans, or tofu with every meal. DH3: Eating vegetables other than kimchi with every meal. DH4: Eating one portion of fruit or drinking one portion of fruit juice every day. DH5: Eating more than two portions of fried or stir-fried food every week. DH6: Eating more than two portions of fatty meat (e.g., bacon, ribs, eel) every week. DH7: Generally adding table salt or soy sauce to food. DH8: Eating three regular meals per day. DH9: Eating ice cream, cake, snacks, and soda (e.g., cola, cider) as snacks more than twice a week. DH10: Eating a variety of food every day. The possible responses to dietary habits (DHs) were “always”, “generally”, or “seldom”. The daily TV-watching time was categorized as <1 h, 1–2 h, and >2 h. Eating breakfast everyday was grouped as yes or no. All of the results were obtained by multiple regression analyses after adjusting for sex, maternal obesity, body mass index at 7 years of age, and household income.

## Supplementary Materials

The following are available online at http://www.mdpi.com/2072-6643/9/1/4/s1, Table S1: Composition of food groups; Table S2: Univariate associations between potential factors and dietary pattern scores at 7 years of age.
