# Supplementary material for: Which Diet-Related Behaviors in Childhood Influence a Healthier Dietary Pattern? From the Ewha Birth and Growth Cohort
Source: Nutrients. 2016 Dec 23;9(1):4. doi: 10.3390/nu9010004 (PMC5295048; doi:10.3390/nu9010004)
Supplement: Supplementary file 1 [file nutrients-09-00004-s001.docx]

Supplementary Materials: Which Diet-Related Behaviors in Childhood Influence a Healthier Dietary Pattern? From the Ewha Birth and Growth Cohort

Hye Ah Lee, Hyo Jeong Hwang, Se Young Oh, Eun Ae Park, Su Jin Cho, Hae Soon Kim and Hyesook Park

**Table S1.** Composition of food groups.

| **Food Groups** | **Food Items** |
| --- | --- |
| Rice | Rice, rice with mixed grain, rice cakes, dried seaweed rolls (*kimbab*), rice cake and dumpling soup (*mandukuk*), sliced rice cake soup (*tokkuk*), cereal, stir-fried rice cake (*[tteokbokki](http://endic.naver.com/enkrEntry.nhn?entryId=188a1a7519394ba78907cb59a54f889e&query=%EB%96%A1%EB%B3%B6%EC%9D%B4)*) |
| Noodles | Korean noodles (*sujebi*)/handmade noodles in broth (*kalguksu*), black bean paste noodles/spaghetti, Korean instant noodles (*Ramyeon*) |
| Bread | Bread/toast, bread (red beans bread, cake etc.) |
| Pizza | Pizza, hamburger |
| Jam | Butter, margarine, jam, mayonnaise |
| Potato | Fried potato, sweet potato, potato |
| Meat | Korean-style blood sausage, offal, beef, rib, pork, ham, sausage, chicken (fried), chicken |
| Eggs | Eggs/quail eggs |
| White fish | Yellow croaker, cutlass fish, pollack, dried pollack, etc. |
| Blue fish | Mackerel, Pacific saury, Japanese Spanish mackerel etc., canned fish (tuna, pacific saury), anchovy |
| Shellfish | Squid, small octopus, shrimp, oyster/shellfish, salted-fermented fish, fish paste |
| Bean | Tofu, soybean curd, braised bean |
| White vegetables | Kimchi (cabbage), kimchi (cubed radish roots), kimchi (radish leaves), kimchi (*nabak*, *dongchimi*) radish, bean sprouts, bean sprout, Napa cabbage, Chinese bellflower/bracken |
| Green vegetables | Lettuce/cabbage, cucumber, spinach, sesame leaves/green pepper, onion |
| Yellow vegetables | Carrot, pumpkin |
| Mushrooms | Mushrooms |
| Seaweed | Laver, brown seaweed |
| Milk | Whole milk, low-fat milk, yogurt, ice cream, cheese, drinking yogurt |
| Soda | Cocoa, soda (cola, cider), soymilk, cinnamon punch (*sujeonggw*a), rice punch (*sikhye*), low-calorie soda (diet cola/cider, zero cola/cider etc.), sports drink |
| Candy | Chocolate, candy, sweet biscuit, cracker/cookie |
| Nuts | Peanuts (pine nuts, walnuts) |
| Fruit | Strawberry, apple, pear, tomato/mini-tomato, peach/plum, mandarin/orange, banana, fruit juice, oriental melon/melon, watermelon, grapes |

**Table S2.** Univariate associations between potential factors and dietary pattern scores at 7 years of age.

|  |  | **Healthy Intake** | | | **Animal Food Intake** | | | **Snack Intake** | | |
| --- | --- | --- | --- | --- | --- | --- | --- | --- | --- | --- |
|  |  | **Mean** | **S.D.** | ***p*** | **Mean** | **S.D.** | ***p*** | **Mean** | **S.D.** | ***p*** |
| Sex | Boys (*n* = 138) | −0.084 | 0.49 | 0.47 | −0.153 | 0.44 | 0.03 | 0.050 | 0.92 | 0.31 |
|  | Girls (*n* = 141) | −0.128 | 0.50 |  | −0.030 | 0.57 |  | −0.055 | 0.80 |  |
| Body mass index (BMI) ^†^ |  | 0.005 | 0.02 | 0.75 | -0.006 | 0.02 | 0.70 | -0.036 | 0.03 | 0.19 |
| Paternal education | Graduated high school (*n* = 42) | −0.202 | 0.47 | 0.16 | 0.006 | 0.38 | 0.18 | −0.148 | 0.64 | 0.20 |
|  | Some college or higher (*n* = 230) | −0.081 | 0.51 |  | −0.103 | 0.50 |  | 0.040 | 0.90 |  |
| Maternal education | Graduated high school (*n* = 55) | −0.154 | 0.45 | 0.40 | −0.129 | 0.41 | 0.48 | −0.005 | 0.77 | 0.89 |
|  | Some college or higher (*n* = 218) | −0.089 | 0.51 |  | −0.078 | 0.50 |  | 0.012 | 0.89 |  |
| Monthly household income | Low (*n* = 56) | −0.190 | 0.45 | 0.03 | −0.072 | 0.45 | 0.95 | 0.040 | 0.93 | 0.08 |
|  | Middle (*n* = 113) | −0.150 | 0.50 | Pt < 0.01 | −0.083 | 0.46 | Pt < 0.59 | −0.121 | 0.67 | Pt = 0.16 |
|  | High (*n* = 103) | 0.003 | 0.51 |  | −0.097 | 0.52 |  | 0.141 | 0.99 |  |
| Paternal obesity | Normal (*n* = 149) | −0.096 | 0.52 | 0.85 | −0.098 | 0.45 | 0.63 | 0.011 | 0.96 | 0.74 |
|  | Obese (*n* = 95) | −0.109 | 0.49 |  | −0.066 | 0.56 |  | 0.050 | 0.78 |  |
| Maternal obesity | Normal (*n* = 221) | −0.089 | 0.52 | 0.58 | −0.094 | 0.49 | 0.66 | 0.047 | 0.92 | 0.10 |
|  | Obese (*n* = 39) | −0.137 | 0.42 |  | −0.057 | 0.41 |  | −0.202 | 0.57 |  |
| Watching TV | <1 h (*n* = 39) | −0.020 | 0.53 | 0.26 | −0.211 | 0.42 | 0.06 | −0.392 | 0.53 | 0.01 |
|  | 1 h to < 2 h (*n* = 109) | −0.095 | 0.55 | Pt = 0.09 | −0.099 | 0.50 | Pt = 0.11 | 0.001 | 0.83 | Pt = 0.02 |
|  | ≥2 h (*n* = 106) | −0.168 | 0.41 |  | −0.004 | 0.49 |  | 0.070 | 0.87 |  |
| Eating breakfast every day | No (*n* = 42) | −0.352 | 0.33 | <0.001 | −0.093 | 0.40 | 0.97 | −0.020 | 0.86 | 0.89 |
|  | Yes (*n* = 235) | −0.059 | 0.51 |  | −0.091 | 0.49 |  | 0.000 | 0.86 |  |
| DH1 | Always (*n* = 67) | 0.107 | 0.54 | < 0.001 | −0.076 | 0.55 | 0.94 | 0.153 | 0.87 | 0.01 |
|  | Generally (*n* = 129) | −0.161 | 0.45 | Pt < 0.01 | −0.099 | 0.46 | Pt = 0.59 | 0.034 | 0.88 | Pt < 0.01 |
|  | Seldom (*n* = 80) | −0.159 | 0.50 |  | −0.099 | 0.43 |  | −0.229 | 0.64 |  |
| DH2 | Always (*n* = 108) | −0.010 | 0.51 | <0.01 | −0.070 | 0.56 | 0.06 | 0.025 | 0.92 | 0.68 |
|  | Generally (*n* = 131) | −0.097 | 0.50 | Pt < 0.01 | −0.063 | 0.42 | Pt = 0.04 | −0.016 | 0.77 | Pt = 0.53 |
|  | Seldom (*n* = 36) | −0.341 | 0.36 |  | −0.264 | 0.30 |  | −0.114 | 0.74 |  |
| DH3 | Always (*n* = 72) | 0.046 | 0.47 | <0.0001 | −0.119 | 0.56 | 0.06 | −0.139 | 0.80 | 0.28 |
|  | Generally (*n* = 123) | −0.026 | 0.53 | Pt < 0.0001 | −0.023 | 0.43 | Pt = 0.23 | 0.005 | 0.76 | Pt = 0.37 |
|  | Seldom (*n* = 81) | −0.329 | 0.37 |  | −0.180 | 0.44 |  | 0.070 | 0.93 |  |
| DH4 | Always (*n* = 130) | 0.016 | 0.48 | <0.001 | −0.104 | 0.54 | 0.44 | 0.088 | 0.91 | 0.09 |
|  | Generally (*n* = 105) | −0.136 | 0.53 | Pt < 0.0001 | −0.053 | 0.43 | Pt = 0.62 | −0.056 | 0.73 | Pt = 0.03 |
|  | Seldom (*n* = 40) | −0.335 | 0.33 |  | −0.161 | 0.32 |  | −0.220 | 0.76 |  |
| DH5 | Always (*n* = 92) | −0.119 | 0.38 | 0.87 | −0.097 | 0.57 | 0.99 | 0.113 | 0.97 | 0.13 |
|  | Generally (*n* = 143) | −0.083 | 0.55 | Pt = 0.97 | −0.092 | 0.42 | Pt = 0.63 | −0.038 | 0.75 | Pt = 0.03 |
|  | Seldom (*n* = 40) | −0.101 | 0.53 |  | −0.087 | 0.41 |  | −0.190 | 0.68 |  |
| DH6 | Always (*n* = 21) | 0.023 | 0.55 | 0.37 | 0.069 | 0.53 | <0.001 | 0.074 | 1.15 | 0.39 |
|  | Generally (*n* = 95) | −0.142 | 0.43 | Pt = 0.96 | 0.026 | 0.45 | Pt < 0.001 | 0.070 | 0.72 | Pt = 0.17 |
|  | Seldom (*n* = 159) | −0.087 | 0.53 |  | −0.185 | 0.46 |  | −0.068 | 0.83 |  |
| DH7 | Always (*n* = 12) | 0.082 | 0.54 | 0.24 | 0.235 | 0.54 | <0.01 | 0.233 | 1.01 | 0.01 |
|  | Generally (*n* = 60) | −0.171 | 0.46 | Pt = 0.60 | 0.034 | 0.55 | Pt < 0.01 | 0.237 | 0.87 | Pt = 0.02 |
|  | Seldom (*n* = 202) | −0.089 | 0.50 |  | −0.147 | 0.43 |  | −0.095 | 0.79 |  |
| DH8 | Always (*n* = 188) | −0.041 | 0.50 | <0.01 | −0.090 | 0.47 | 0.78 | −0.037 | 0.80 | 0.31 |
|  | Generally (*n* = 80) | −0.185 | 0.47 | Pt < 0.01 | −0.090 | 0.48 | Pt = 0.77 | 0.075 | 0.91 | Pt = 0.96 |
|  | Seldom (*n* = 8) | −0.520 | 0.18 |  | −0.210 | 0.31 |  | −0.344 | 0.51 |  |
| DH9 | Always (*n* = 82) | −0.155 | 0.46 | 0.28 | −0.019 | 0.50 | 0.15 | 0.135 | 0.83 | 0.01 |
|  | Generally (*n* = 105) | −0.040 | 0.53 | Pt = 0.40 | −0.095 | 0.46 | Pt = 0.17 | 0.059 | 0.82 | Pt < 0.01 |
|  | Seldom (*n* = 88) | −0.107 | 0.48 |  | −0.162 | 0.46 |  | −0.227 | 0.78 |  |
| DH10 | Always (*n* = 77) | 0.124 | 0.50 | <0.0001 | −0.021 | 0.56 | 0.26 | −0.048 | 0.81 | 0.68 |
|  | Generally (*n* = 125) | −0.098 | 0.50 | Pt < 0.0001 | −0.135 | 0.45 | Pt = 0.14 | 0.034 | 0.87 | Pt = 0.61 |
|  | Seldom (*n* = 74) | −0.319 | 0.36 |  | −0.095 | 0.41 |  | −0.058 | 0.77 |  |

Pt: *p*-value for trend. S.D. = standard deviation. DH1: Eating more than two portions of milk or dairy products every day. DH2: Eating meat, fish, egg, beans, or tofu with every meal. DH3: Eating vegetables other than kimchi with every meal. DH4: Eating one portion of fruit or drinking one portion of fruit juice every day. DH5: Eating more than two portions of fried or stir-fried food every week. DH6: Eating more than two portions of fatty meat (e.g., bacon, ribs, eel) every week. DH7: Generally adding table salt or soy sauce to food. DH8: Eating three regular meals per day. DH9: Eating ice cream, cake, snacks, and soda (e.g., cola, cider) as snacks more than twice a week. DH10: Eating a variety of food every day. ^†^ Result presented as coefficient with standard error, which was obtained from simple linear regression.
